# Supplementary material for: Integration of 3D-printed cerebral cortical tissue into an ex vivo lesioned brain slice
Source: Nat Commun. 2023 Oct 4;14:5986. doi: 10.1038/s41467-023-41356-w (PMC10551017; doi:10.1038/s41467-023-41356-w)
Supplement: Supplementary file 1 — Supplementary Information [file 41467_2023_41356_MOESM1_ESM.pdf]

Supplementary Information

Integration of 3D-Printed Cerebral Cortical Tissue into an *ex vivo* Lesioned Brain Slice

Yongcheng Jin[1], Ellina Mikhailova[1], Ming Lei[2], Sally A. Cowley[3], Tianyi Sun[2], Xingyun Yang[1], Yujia Zhang[1], Kaili Liu[4], Daniel Catarino da Silva[4], Luana Campos Soares[4], Sara Bandiera[4], Francis G. Szele[4]*, Zoltán Molnár[4]*, Linna Zhou[1,5]* and Hagan Bayley[1]*

[1]Department of Chemistry, University of Oxford, Oxford, OX1 3TA, United Kingdom.

[2]Department of Pharmacology, University of Oxford, Oxford, OX1 3QT, United Kingdom.

[3]James and Lillian Martin Centre for Stem Cell Research, Sir William Dunn School of Pathology, University of Oxford, South Parks Road, Oxford, OX1 3RE, United Kingdom.

[4]Department of Physiology, Anatomy and Genetics, University of Oxford, Oxford, OX1 3PT, United Kingdom.

[5]Ludwig Institute for Cancer Research, Nuffield Department of Medicine, University of Oxford, Oxford, OX3 7DQ, United Kingdom.

*E-mail: francis.szele@dpag.ox.ac.uk, zoltan.molnar@dpag.ox.ac.uk, linna.zhou@chem.ox.ac.uk and hagan.bayley@chem.ox.ac.uk

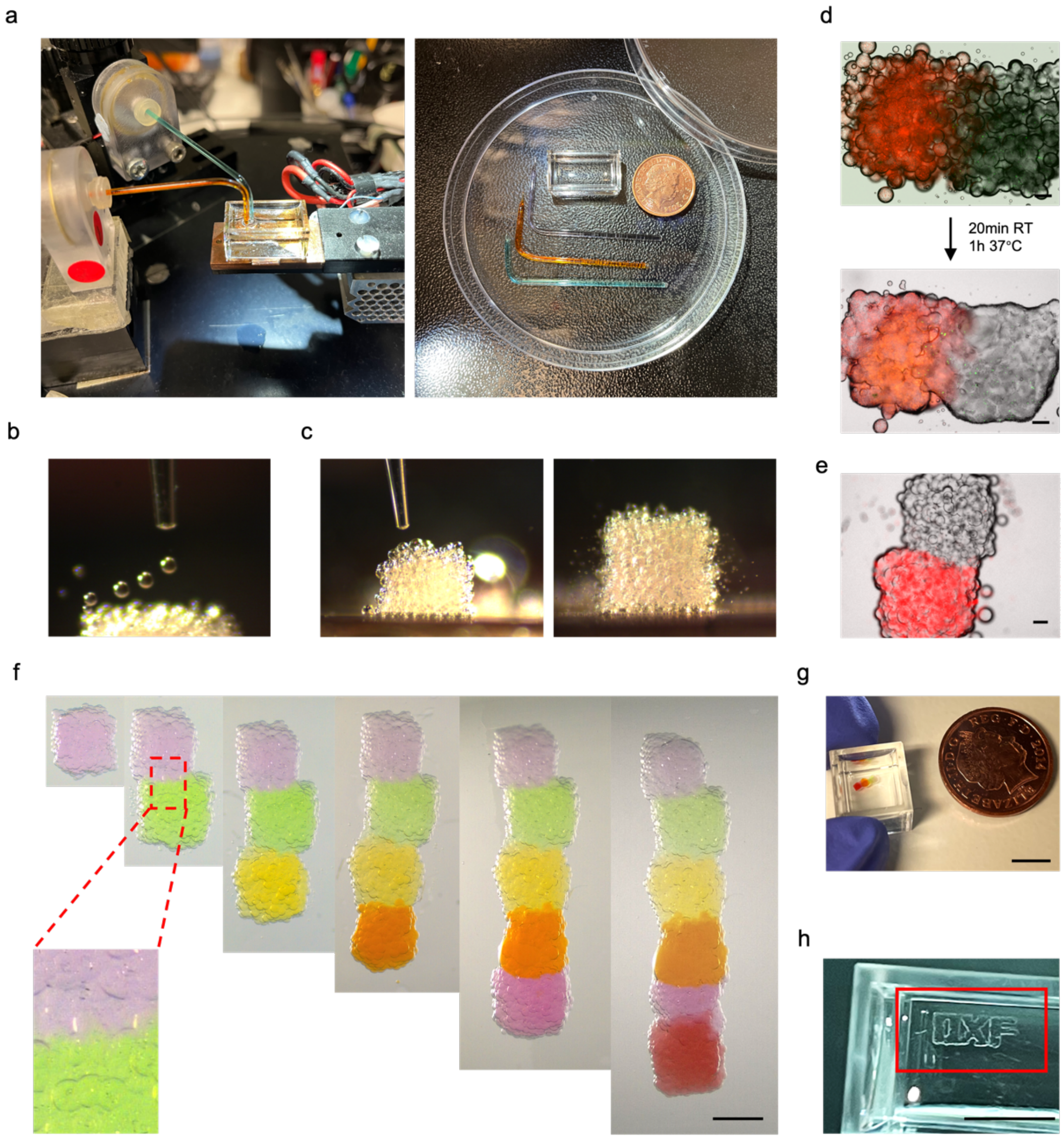

Supplementary Fig. 1: Droplet-based 3D printing. a. The droplet-based 3D bioprinter (left) and various components (right) including the glass printing cuvette and printing nozzles in comparison to a ten-pence coin. **b.** Side-view during the printing process. **c.** Side-views of printed droplet networks containing Matrigel only. **d.** Fluorescence images of a two-layered droplet network containing RFP-labelled UNPs and unlabelled DNPs. Raising the temperature, from room to physiological, facilitated gelation and annealing of printed two-layer networks. **e.** Fluorescence image of two-layered droplet network with fluorescent microbeads in one layer. **f.** Sequential generation of six-layered network by the droplets containing food dye coloured DPBS. Scale bar, 1000 µm. **g.** View of the six-layered network in **'f'**, in comparison to a ten-pence coin. **h.** A magnified view of the droplet network in Fig. 1**l**. The network was printed as 'OXF' and is indicated by the red box. For **'d'** and **'e'**: scale bar, 200 µm. For **'g'** and **'h'**: scale bar, 500 µm.

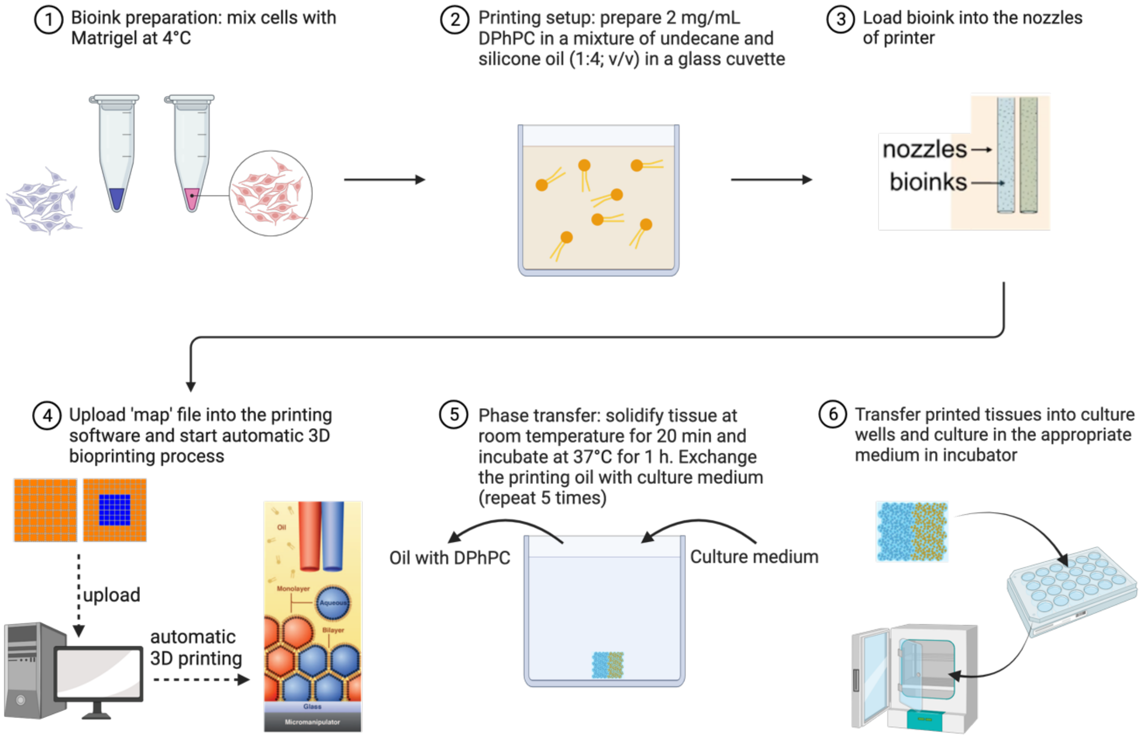

Supplementary Fig. 2: A flow chart of the droplet-based 3D bioprinting process[1-3].

a

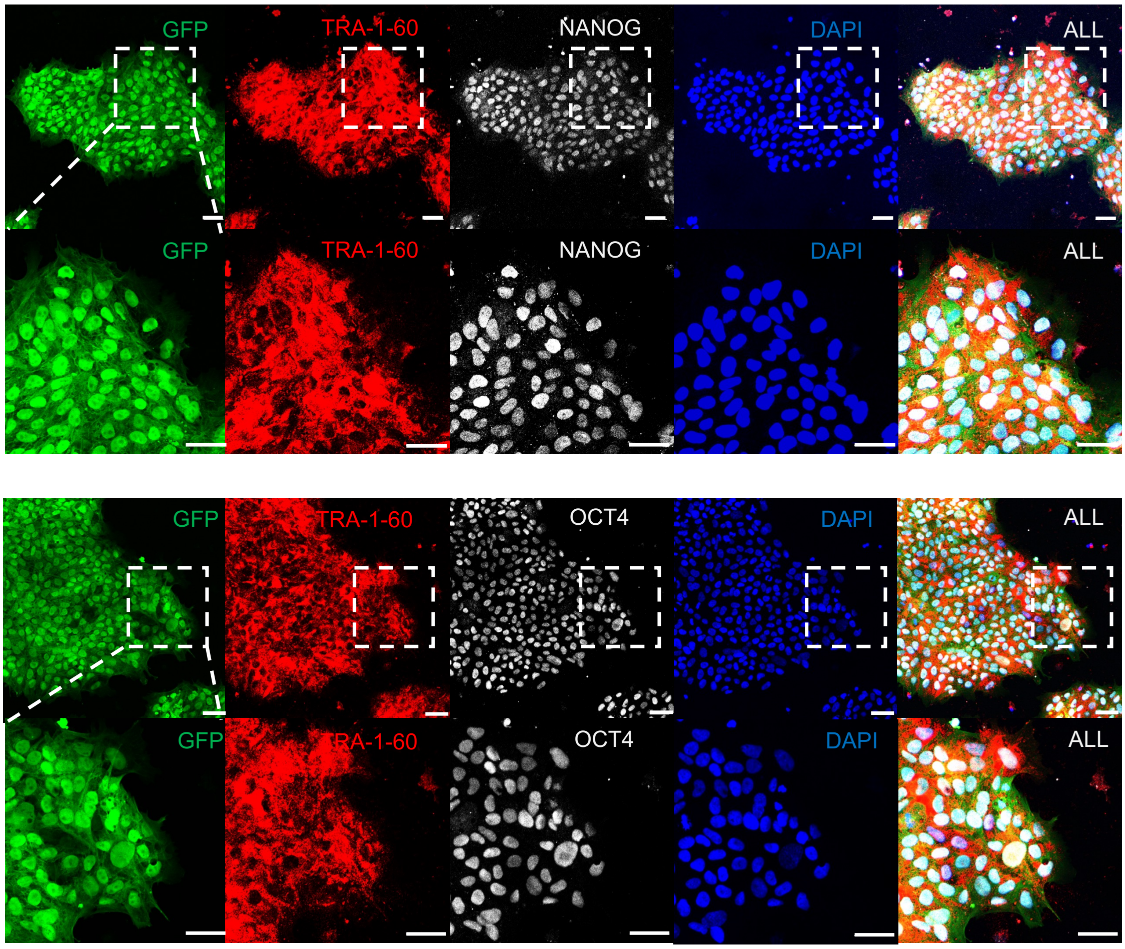

Supplementary Fig. 3: Characterisation of human induced pluripotent stem cells (hiPSCs). a. Confocal fluorescence images of immunostained GFP-labelled hiPSCs showing the expression of pluripotent stem-cell markers TRA-1-60, NANOG and OCT4 in majority of the cells. Images at higher magnification of the regions indicated by the dashed boxes are shown in the second row. Scale bar, 50 µm.

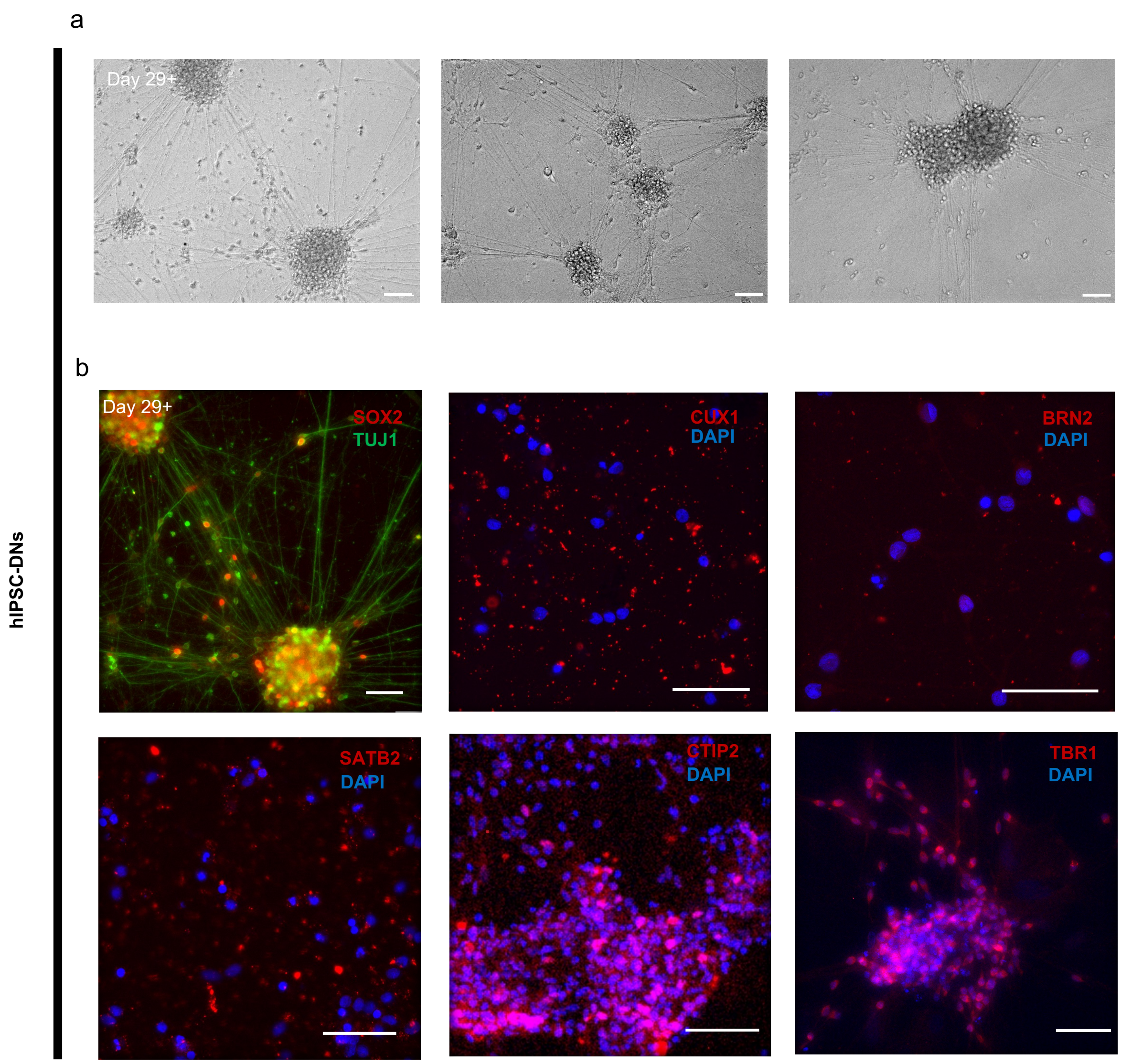

Supplementary Fig. 4: Characterisation of hiPSCs derived deep-layer neurons (hiPSCs-DNs). a. Bright-field images of DIV29+ unlabelled hiPSCs-DNs showing mature neural morphology. **b.** Immunostaining of unlabelled DNs showing expression of the neural stem cell marker SOX2, the general young neuron markers TUJ1 and the deep-layer markers (CTIP2 and TBR1). Expression of upper-layer markers (CUX1 and BRN2) and the middle-upper-layer marker (SATB2) were not detected despite high fluorescence intensity was used to reveal the background. For all panels: scale bar, 50 µm.

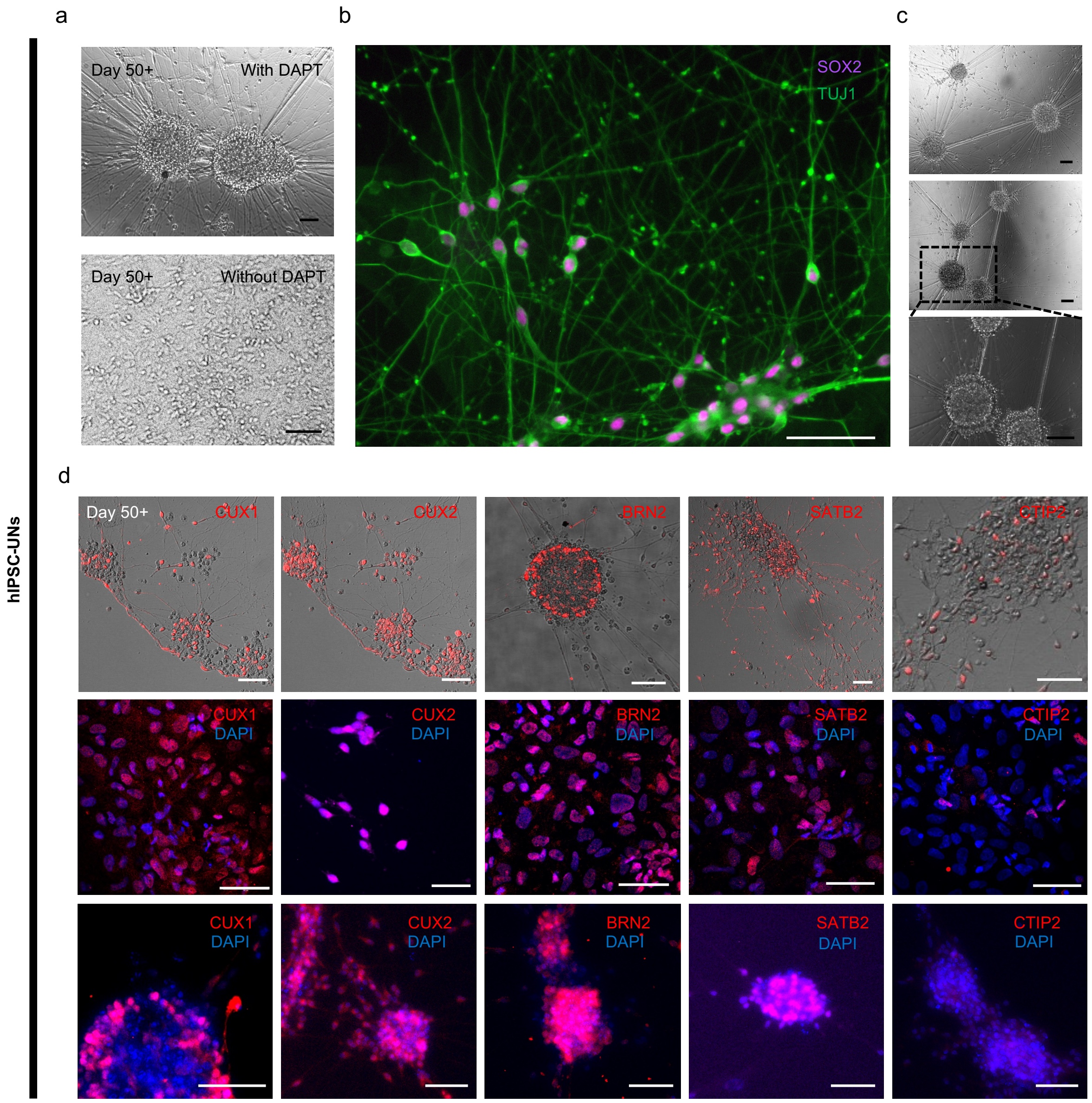

Supplementary Fig. 5: Characterisation of hiPSC-derived upper-layer neurons (hiPSCs-UNs). a. Bright-field images of DIV 50+ RFP-labelled hiPSCs-UNs demonstrating the neuronal morphologies with (top) and without (bottom) DAPT treatment during maturation. UNs matured in NTM with DAPT are shown in **b-d**. **b.** DIV 50+ RFP-labelled UNs immunostained with the neural stem cell marker SOX2 and the general young neuron marker TUJ1. **c.** Bright-field images RFP-labelled hiPSCs-UNs showing mature morphology as indicated by extensive process outgrowth. **d.** Fluorescence images of DIV 50+ RFP-labelled UNs from three independent experiments on each row showing expression of upper-layer markers (CUX1, CUX2 and BRN2) and the middle-upper-layer marker (SATB2), but low expression of the deep-layer marker (CTIP2). For all panels: scale bar, 50 μm.

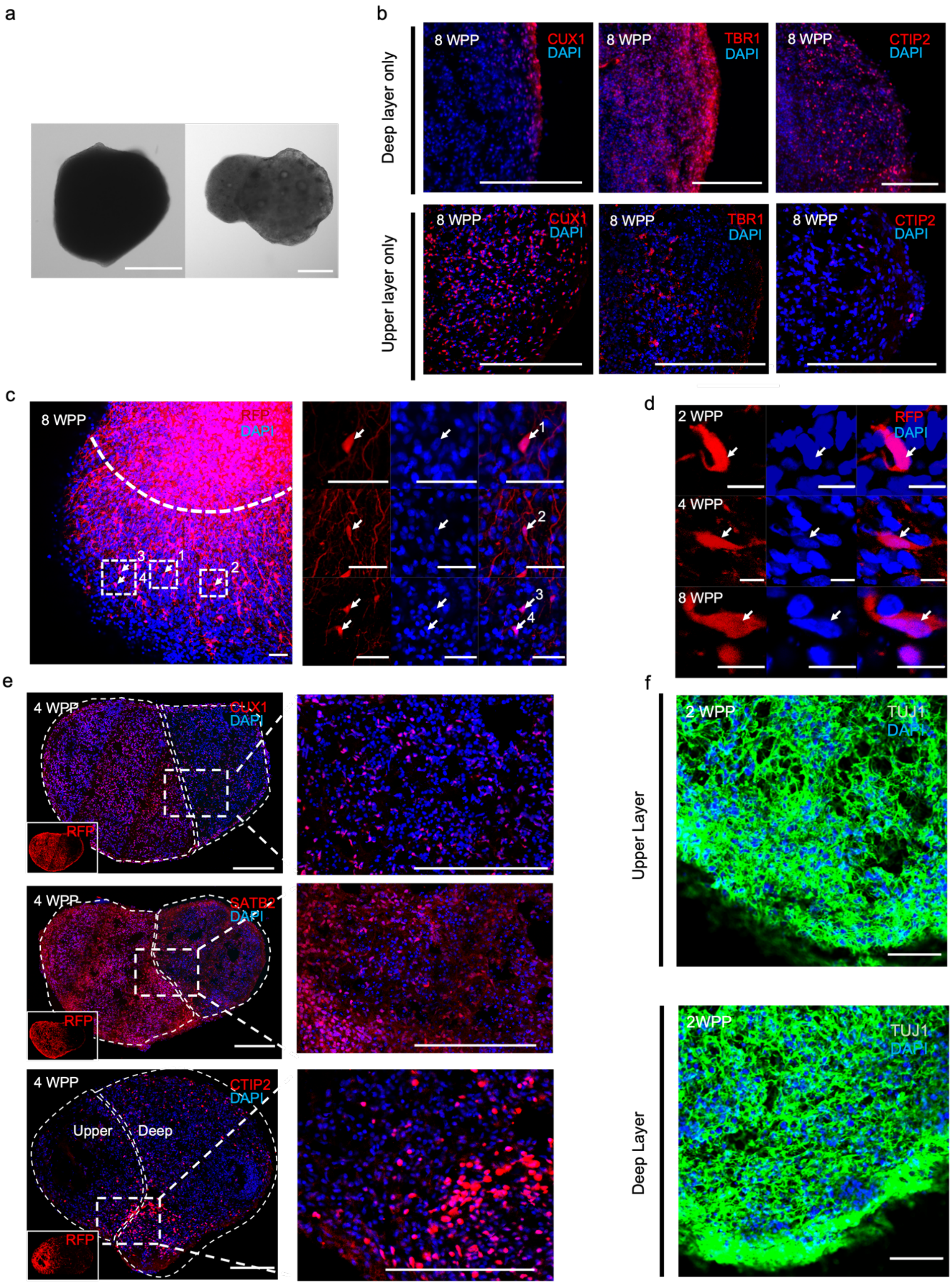

a
b
Deep layer only
Upper layer only
8 WPP
CUX1
DAPI
TBR1
CTIP2
c
8 WPP
RFP
DAPI
d
2 WPP
4 WPP
8 WPP
e
4 WPP
SATB2
Upper
Deep
f
2 WPP
2WPP
TUJ1
Upper Layer
Deep Layer

Supplementary Fig. 6: Further characterisation of droplet-printed cerebral cortical tissues. a. Bright-field images of a 2 WPP deep-layer only (with unlabelled DNs, left) and a two-layer (with unlabelled DNs and RFP-labelled UNs, right) cortical tissues. **b.** Fluorescence images of sectioned 8 WPP unlabelled deep-layer only cortical tissues (top) and RFP-labelled upper-layer only cortical tissues (bottom) showing the expression of the deep-layer markers (CTIP2 & TBR1) and upper-layer marker (CUX1). **c.** Confocal z-projection image (left) and high magnification images (right) showing cross-layer neuron migration in printed two-layer tissue at 8 WPP, visualized by RFP expression in UNs and DAPI nuclear staining in both RFP-labelled UNs and unlabelled DNs. Dashed boxes indicate the magnified regions. Arrows and numbers indicate migrating neurons. Scale bar, 50 μm. **d.** Confocal images of 30 μm-thickness sections of 2, 4 and 8 WPP two-layer tissues with RFP-labelled UNs and unlabelled DNs showing cross-layer neuron migration, visualized by RFP and DAPI co-localisation. Scale bar, 10 μm. **e.** Immunofluorescence images at 4WPP of sectioned two-layer tissues showing expression of the layer-specific markers (CUX1, SATB2 and CTIP2). Bottom left small image shows RFP expression in RFP-labelled UNs of the tissue. Dashed lines outline the layers and dashed boxes indicate the magnified areas. **f.** Confocal images of sectioned two-layer tissues showing the expression of young neuronal marker TUJ1. Scale bar, 50 μm. For panels **'a'**, **'b'** & **'e'**: scale bar, 200 μm.

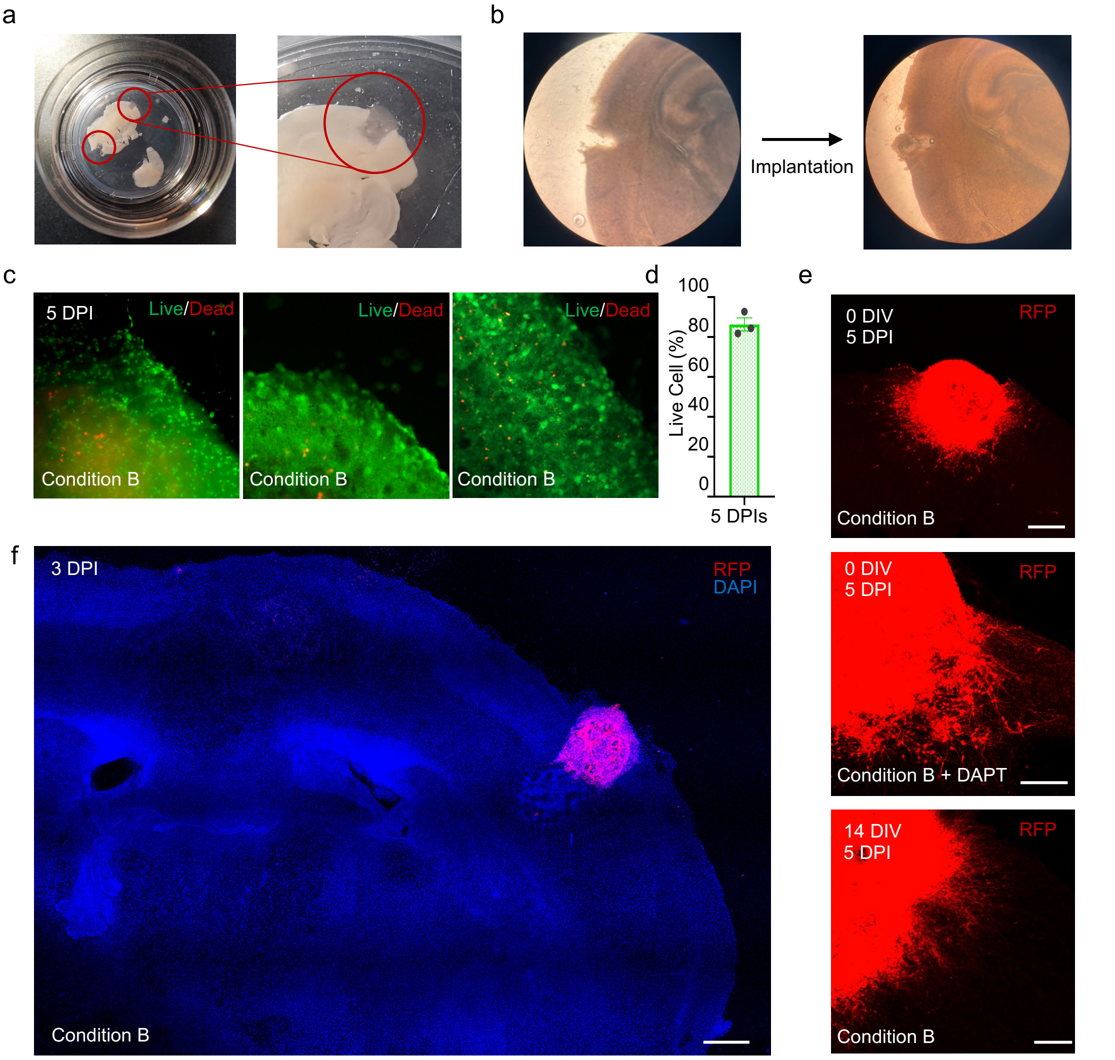

Supplementary Fig. 7: Characterisation of implanted mouse brain explants. a. 0 DPI explant with a lesion in the left cerebral hemisphere and a lesion implanted with printed unlabelled deep-layer tissue in the right cerebral hemisphere. Right, a magnified image of lesion on the right hemisphere implanted with printed deep-layer cortical tissue. **b.** A bright-field image of a 0 DPI explant with a lesion implanted with a printed unlabelled deep-layer cortical tissue. **c.** Fluorescence images of a live/dead assay of RFP-labelled deep-layer cortical tissue implanted explant cultured under condition B at 5 DPIs. **d.** Quantitative live/dead analysis of host cells of 5 DPIs at condition B (n = 3). **e.** Further examples of implanted RFP-labelled deep-layer tissues under different nutrient conditions and with different pre-implantation culture times. **f.** Tiled fluorescence confocal image of an explant implanted with a two-layer printed tissue in the right hemisphere. Cells were visualized by RFP (UNs) and DAPI nuclear staining in RFP-labelled UNs, unlabelled DNs and the host. For all panels: scale bar, 200µm.

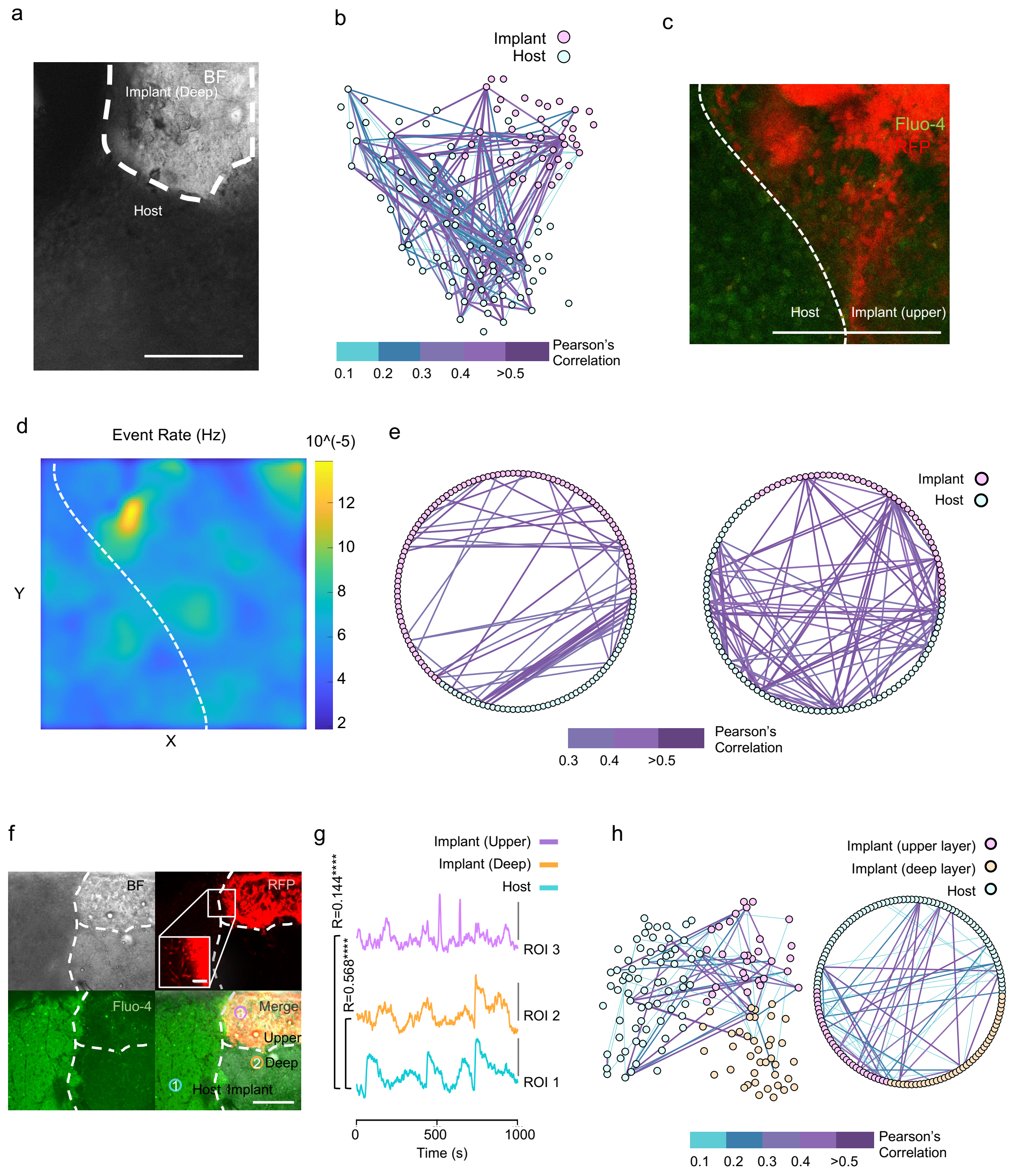

Supplementary Fig. 8: Functional analysis of implanted mouse brain explants. **a.** Bright-field image at 5 DPIs of an explant implanted with unlabelled deep-layer cortical tissue (as indicated in **'Fig. 5h-j')**. The contrast difference between the implant and host marks the border between them. **b.** Network analysis of firing-correlated neurons between the implant and the host in **'Supplementary Fig.8a'** on 5 DPI. Circles correspond to neurons and lines indicate correlated firings. **c.** Fluorescence image of an explant implanted with RFP-labelled upper-layer tissue, as also indicated on **'Fig. 5k-m'**. The RFP-labelled UNs and Fluo-4 labelled implant and host tissue mark the implant-host interface. **d.** Heatmap of neuron firing rate showing comparable neuron activity between explant and the implanted tissue. **e.** Network analysis of firing-correlated neurons for the 5 DPI implanted explants found in **'Fig.5h' (Left)** and **'Fig.5k' (Right)** in a circular layout. **f.** Confocal images of an explant implanted with two-layer tissue containing RFP-labelled UNPs and unlabelled DNPs at 5 DPI. The dashed lines show the interfaces between the upper

layer of the implant, the deep layer of the implant and the host. The solid boxes show a magnified view of RFP-labelled upper layer of the implant with a strong contrast, demonstrating migrating RFP-labelled neurons. Scale bar in the box, 50 μm. **g.** Single-cell calcium traces, colour-coded ROIs as indicated in '**f**'. Scale bar: $\Delta F/F_0$ = 0.05; R: Pearson's correlation value; and P-value of Pearson's correlation: ****, $P < 0.0001$. **h.** Correlated neuron pairs within '**f**', among the upper layer of the implant, the deep layer of the implant and the host at 5 DPI by network analysis in conventional (left) and circular layout (right). Points representing neurons and the lines between them indicate correlated calcium signals. For all panels: scale bar, 200μm.

Supplementary Table 1. Culture Medium Formula

Neural Induction Medium (NIM) 100mL					
**Item**	**Volume**	**Final Conc**	**Stock Conc**	**Supplier**	**Cat no**
DMEM/F12 Medium	~49 mL	NA	1X	Life Technologies	21331020
Neurobasal Medium	~49 mL	NA	1X	Life Technologies	21103-049
B27 supplement	1 mL	NA	NA	Life Technologies	17504044
N2 supplement	0.5 mL	NA	NA	Life Technologies	17502-048
GlutaMax	1 mL	NA	100X	Life Technologies	35050-038
LDN193189	10 µL	100nM	1 mM	Sigma	SML0559
SB431542	100 µL	10 µM	10 mM	Cambridge Bioscience	ZRD-SB-50
Puromycin (opt)	50 µL	2.5µg/ml	5 mg/ml	MP Biomedicals UK	210055225
**Neural Maintenance Medium (NMM) 100mL**					
**Item**	**volume**	**Final Conc**	**Stock Conc**	**Supplier**	**Cat no**
DMEM/F12 Medium	~49 mL	NA	1X	Life Technologies	21331020
Neurobasal Medium	~49 mL	NA	1X	Life Technologies	21103-049
B27 supplement	1 mL	NA	NA	Life Technologies	17504044
N2 supplement	0.5 mL	NA	NA	Life Technologies	17502-048
GlutaMax	1 mL	NA	100X	Life Technologies	35050-038
Puromycin (opt)	50 µL	2.5µg/ml	5 mg/ml	MP Biomedicals UK	210055225
**Neural Maintenance Medium + Growth Factors (NMM + GFs) 100mL**					
**Item**	**volume**	**Final Conc**	**Stock Conc**	**Supplier**	**Cat no**
DMEM/F12 Medium	~49 mL	NA	1X	Life Technologies	21331020
Neurobasal Medium	~49 mL	NA	1X	Life Technologies	21103-049
B27 supplement	1 mL	NA	NA	Life Technologies	17504044
N2 supplement	0.5 mL	NA	NA	Life Technologies	17502-048
GlutaMax	1 mL	NA	100X	Life Technologies	35050-038
Fibroblast Growth Factor-2 (FGF-2)	10 µL	10 ng/mL	100µg/mL	R&D Systems	4114-TC-01M
Epidermal Growth Factor (EGF)	10 µL	10 ng/mL	100µg/mL	Life Technologies	PHG0311
Brain-derived Neurotrophic Factor (BDNF)	10 µL	10 ng/mL	100µg/mL	Life Technologies	PHC7074
**Freezing Medium 10 mL**					
**Item**	**volume**	**Final Conc**	**Stock Conc**	**Supplier**	**Cat no**
ESC-qualified FBS	9 mL	NA	NA	Gibco	16141061
DMSO	1 mL	NA	NA	Merck	D2650-100ML
**Neural Terminal Medium (NTM) 100 mL**					
**Item**	**volume**	**Final Conc**	**Stock Conc**	**Supplier**	**Cat no**
Neurobasal Medium	97 mL	NA	1X	Life Technologies	21103-049
B27 supplement	2 mL	NA	NA	Life Technologies	17504044
GlutaMax	1 mL	NA	100X	Life Technologies	35050-038
DAPT	10 µL	10 µM	100 mM	Tocris	Oct-34
Puromycin (opt)	50 µL	2.5µg/ml	5 mg/ml	MP Biomedicals UK	210055225
**Brain Explant Culture Medium Condition A 100mL**					
**Item**	**volume**	**Final Conc**	**Stock Conc**	**Supplier**	**Cat no**
DMEM/F12 Medium	~36 mL	NA	1X	Life Technologies	21331020
Neurobasal Medium	~36 mL	NA	1X	Life Technologies	21103-049
B27 supplement	1 mL	NA	NA	Life Technologies	17504044
N2 supplement	0.5 mL	NA	NA	Life Technologies	17502-048
GlutaMax	1 mL	NA	100X	Life Technologies	35050-038
Pen/Strep	1 mL	NA	10,000 U/mL	Gibco	15140122
Horse Serum	25 mL	NA	NA	Life Technologies	16050130
**Brain Explant Culture Medium Condition B 100mL**					
**Item**	**volume**	**Final Conc**	**Stock Conc**	**Supplier**	**Cat no**
Brainphys Neural Medium	72.5 mL	NA	NA	Stemcell Technologies	5792
SM1 supplement	1.5 mL	NA	NA	Stemcell Technologies	5792
Pen/Strep	1 mL	NA	10,000 U/mL	Gibco	15140122
Horse Serum	25 mL	NA	NA	Life Technologies	16050130

Supplementary Table 2. Consumables

Consumables (Cell Culture)					
**Item**	**volume**	**Final Conc**	**Stock Conc**	**Supplier**	**Cat no**
mTeSR Plus Medium	NA	NA	NA	Stemcell Technologies	100-0276
Geltrex	NA	NA	NA	Gibco	A1413302
StemPro Accutase	NA	NA	NA	Life Technologies	A1110501
DPBS	NA	NA	NA	Gibco	14190144
UltraPure 0.5M EDTA	NA	0.5 mM	0.5 M	Life Technologies	15575020
Distilled Water	NA	NA	NA	Life Technologies	15230089
Y-27632	NA	10 µM	1mM	Abcam	ab120129-10mg
ReLeSR	NA	NA	NA	Stemcell Technologies	5872
**Consumables (Droplet Printing)**					
**Item**	**volume**	**Final Conc**	**Stock Conc**	**Supplier**	**Cat no**
Silicone oil AR20	NA	NA	NA	Sigma	10836
Undecane	NA	NA	NA	Sigma	1120-21-4
DPHPC	NA	NA	NA	Avanti	850356
Trimethoxysilane	NA	5% v/v	NA	Sigma	281778
Matrigel	NA	NA	NA	Corning	354230
**Consumables (Brain Explant)**					
**Item**	**volume**	**Final Conc**	**Stock Conc**	**Supplier**	**Cat no**
EBSS	NA	NA	NA	Life Technologies	24010043
Culture Insert	NA	NA	NA	Merck	PICM0RG50
X30 Cell Imaging Dish,	NA	NA	NA	Fisher Scientific UK	15670537
BrainPhys Imaging Optimized Medium	NA	NA	NA	Stemcell Technologies	5796
UltraPure Low Melting Point Agarose	NA	NA	NA	Life Technologies	16520050
Microtome blade	NA	NA	NA	Fisher Scientific	11912355
Needle	NA	NA	NA	Fisher Scientific	10749891
Super Glue	NA	NA	NA	Office Depot	4086446
**Consumables (qPCR)**					
**Item**	**volume**	**Final Conc**	**Stock Conc**	**Supplier**	**Cat no**
LunaScript(R) RT SuperMix Kit	NA	NA	NA	New England Biolabs	E3010L
Monarch(R) Total RNA Miniprep Kit	NA	NA	NA	New England Biolabs	T2010S
Luna(R) Universal qPCR Master Mix	NA	NA	NA	New England Biolabs	M3003L
MicroAmp Fast Optical 96-Well Reaction Plate	NA	NA	NA	Life Technologies	4346906
Nuclease-Free water	NA	NA	NA	QIAGEN	129114
**Consumables (Immunostaining, live/dead assay and Fluo-4 imaging)**					
**Item**	**volume**	**Final Conc**	**Stock Conc**	**Supplier**	**Cat no**
Ibidi µ-Slide 18 Well	NA	NA	NA	ThistleScientific	SKU 81816
Paraformaldehyde 4%	NA	4%	4%	Alfa Aesar	J61899.AK
Glycine 1 M Solution	NA	NA	NA	Merck	67419-1ML-F
Triton X-100	NA	NA	NA	Merck	93443-100ML
Tween-20	NA	NA	NA	Alfa Aesar	P9416-50ML
Normal Goat Serum	NA	NA	NA	Abcam	ab7481
Normal Donkey Serum	NA	NA	NA	Abcam	ab7475
DAPI Solution	NA	1X	10000X	Merck	MBD0015-1ML
Mounting Medium With DAPI	NA	1X	1X	Abcam	ab104139
Fluo-4 Direct Calcium Assay Kit	NA	1X	2X	Life Technologies	F10471
Calcein-AM	NA	2.5 µM	NA	Cambridge bioscience	1755-50
Propidium iodide	NA	5.0 µM	NA	Sigma	P4170
**Plastics and others**					
1.8ml Cryogenic Vial	NA	NA	NA	STARLAB	E3090-6222
10µl Pipette Tip	NA	NA	NA	STARLAB	S1121-2710
20µl Pipette Tip	NA	NA	NA	STARLAB	S1120-1710
200µl Pipette Tip	NA	NA	NA	STARLAB	S1126-7810
1000µl Pipette Tip	NA	NA	NA	STARLAB	S1120-8810
6 Well Tissue Culture Plate	NA	NA	NA	Greiner Bio-One	657160
12 Well Tissue Culture Plate	NA	NA	NA	Greiner Bio-One	665180
24Well Tissue Culture Plate	NA	NA	NA	Greiner Bio-One	662160
48 Well Tissue Culture Plate	NA	NA	NA	Greiner Bio-One	677180
96 well Tissue Culture Plate	NA	NA	NA	Greiner Bio-One	655180
96 well Assessment Plate	NA	NA	NA	Corning	CLS3603
5mL Stripette	NA	NA	NA	Scientific Laboratory Supp	4487
10mL Stripette	NA	NA	NA	Scientific Laboratory Supp	4488
25mL Stripette	NA	NA	NA	Scientific Laboratory Supp	4489
Cryo Container	NA	NA	NA	VWR International	479-3200
Microslides	NA	NA	NA	VWR International	631-0448
PAP Pen	NA	NA	NA	Merck	Z672548-1EA

Supplementary Table 3. Antibodies and Primers

Primary Antibodies				
**Target**	**Original Species**	**Manufacturer**	**Dilution Factor**	**Cat. No**
CUX1 (Ext.Data Fig. 4d top row)	Ms	AbCam	100	ab54583
CUX1	Rb	Santa Cruz Biotechnology	100	sc-13024
CUX2	Rb	AbCam	200	ab216588
BRN2	Ms	Santa Cruz Biotechnology	100	sc-393324
SATB2	Rb	AbCam	200	ab92446
CTIP2	Rat	AbCam	200	ab18465
TBR1	Rb	Merck	500	AB10554
SOX2	Rb	Millipore	100-200	ab5603
TUJ1	Ms	AbCam	500-1000	ab78078
GFAP	Rat	Invitrogen	200	13-0300
HNCAM	Rb	AbCam	200	ab75813

Secondary Antibodies					
**Target Species**	**Original Species**	**Fluorophore**	**Manufacturer**	**Dilution Factor**	**Cat. No**
Rb	Goat	Alex488	Invitrogen	1000	a11006
Ms	Goat	Alex488	Invitrogen	1000	a32723
Ms	Goat	Alex633	Invitrogen	1000	a21052
Rat	Goat	Alex647	Invitrogen	1000	a21247
Rb	Goat	Alex647	Invitrogen	1000	a21245

qPCR Primer			
**Target**	**Forward or Backward**	**5'-3' Sequence**	**Manufacturer**
**PAX6**	**F**	GCCAGCAACACACCTAGTCA	Life Technologies
	**R**	TGTGAGGGCTGTGTCTGTTC	Life Technologies
**Nestin**	**F**	GGAAGAGAACCTGGGAAAGG	Life Technologies
	**R**	CTTGGTCCTTCTCCACCGTA	Life Technologies
**CTIP2**	**F**	GAGTACTGCGGCAAGGTGTT	Life Technologies
	**R**	TAGTTGCACAGCTCGCACTT	Life Technologies
**BRN2**	**F**	GACCTTTGCAGGCGAGTAAC	Life Technologies
	**R**	TCAGGAAGCTGCATTTTGTG	Life Technologies
**CUX1**	**F**	GCTCTCATCGGCCAATCACT	Life Technologies
	**R**	TCTATGGCCTGCTCCACGT	Life Technologies
**CUX2**	**F**	AAGGAGATCGAGTCGCAGAA	Life Technologies
	**R**	CTCCAGGATGCTCTTGATGG	Life Technologies
**18S**	**F**	GAGGATGAGGTGGAACGTGT	Life Technologies
	**R**	TCTTCAGTCGCTCCAGGTCT	Life Technologies

Supplementary References

1 Villar, G., Graham, A. D. & Bayley, H. A tissue-like printed material. *Science* **340**, 48-52, doi:10.1126/science.1229495 (2013).

2 Krishna Kumar, R. *et al.* Droplet printing reveals the importance of micron-scale structure for bacterial ecology. *Nature Communications* **12**, 857, doi:10.1038/s41467-021-20996-w (2021).

3 Zhou, L. *et al.* Lipid-Bilayer-Supported 3D Printing of Human Cerebral Cortex Cells Reveals Developmental Interactions. *Advanced Materials* **32**, 2002183, doi:https://doi.org/10.1002/adma.202002183 (2020).
