# Supplementary material for: Integration of 3D-printed cerebral cortical tissue into an ex vivo lesioned brain slice
Source: Nat Commun. 2023 Oct 4;14:5986. doi: 10.1038/s41467-023-41356-w (PMC10551017; doi:10.1038/s41467-023-41356-w)
Supplement: Supplementary file 3 — Description of Additional Supplementary Files Document [file 41467_2023_41356_MOESM3_ESM.pdf]

## Description of Additional Supplementary Files

**Supplementary Video 1,** 3DReconstructed confocal z-projection image showing cross-layer process outgrowth and neuron migration in a printed two-layer tissue at 8 WPP, visualized by RFP (false coloured as fire) expression in UNs and DAPI nucleus staining in both UN and DNs. Scale bars: 500 µm.

**Supplementary Video 2**, Fluo-4 calcium ion activity recording of the explant implanted with DNPs only at 5 DPI, as indicated in 'Fig. 5h'. Scale bars: 200 µm.
