# Supplementary material for: Integration of 3D-printed cerebral cortical tissue into an ex vivo lesioned brain slice
Source: Nat Commun. 2023 Oct 4;14:5986. doi: 10.1038/s41467-023-41356-w (PMC10551017; doi:10.1038/s41467-023-41356-w)
Supplement: Supplementary file 6 — Reporting Summary [file 41467_2023_41356_MOESM6_ESM.pdf]

nature portfolio

Corresponding author(s): Linna Zhou

Last updated by author(s): Jul 28, 2023

Reporting Summary

Nature Portfolio wishes to improve the reproducibility of the work that we publish. This form provides structure for consistency and transparency in reporting. For further information on Nature Portfolio policies, see our Editorial Policies and the Editorial Policy Checklist.

Statistics

For all statistical analyses, confirm that the following items are present in the figure legend, table legend, main text, or Methods section.

n/a	Confirmed	
☐	☒	The exact sample size (*n*) for each experimental group/condition, given as a discrete number and unit of measurement
☐	☒	A statement on whether measurements were taken from distinct samples or whether the same sample was measured repeatedly
☐	☒	The statistical test(s) used AND whether they are one- or two-sided<br>*Only common tests should be described solely by name; describe more complex techniques in the Methods section.*
☐	☒	A description of all covariates tested
☐	☒	A description of any assumptions or corrections, such as tests of normality and adjustment for multiple comparisons
☐	☒	A full description of the statistical parameters including central tendency (e.g. means) or other basic estimates (e.g. regression coefficient) AND variation (e.g. standard deviation) or associated estimates of uncertainty (e.g. confidence intervals)
☐	☒	For null hypothesis testing, the test statistic (e.g. *F*, *t*, *r*) with confidence intervals, effect sizes, degrees of freedom and *P* value noted<br>*Give P values as exact values whenever suitable.*
☒	☐	For Bayesian analysis, information on the choice of priors and Markov chain Monte Carlo settings
☒	☐	For hierarchical and complex designs, identification of the appropriate level for tests and full reporting of outcomes
☒	☐	Estimates of effect sizes (e.g. Cohen's *d*, Pearson's *r*), indicating how they were calculated

Our web collection on statistics for biologists contains articles on many of the points above.

Software and code

Policy information about availability of computer code

Data collection	Confocal microscopes: Leica LSM780, LSM980 and Leica SP5.<br>Epi-fluorescent microscopes: Leica DMI 8 and Nikon Eclipse Ni-E.<br>RT-qPCR: ProFlex PCR System from Applied Biosystem.
Data analysis	Graphpad Prism (v9.0.1): ploting and statistical analysis.<br>Image J (v2.1.0/1.54c): epi- and confocal image analysis, cell counting, process outgrowth distance and migration analysis.<br>Microsoft Excel (v16.66): data handling and correlation analysis.<br>Rstudio (v1.3.1093) with R package igraph (v1.3.2): correlation analysis and plotting.<br>NETCAL (version 8.4.1): similarity matrix<br>Microsoft Powerpoint (v16.66): figure preparation.

For manuscripts utilizing custom algorithms or software that are central to the research but not yet described in published literature, software must be made available to editors and reviewers. We strongly encourage code deposition in a community repository (e.g. GitHub). See the Nature Portfolio guidelines for submitting code & software for further information.

Data

Policy information about availability of data

All manuscripts must include a data availability statement. This statement should provide the following information, where applicable:

- Accession codes, unique identifiers, or web links for publicly available datasets
- A description of any restrictions on data availability
- For clinical datasets or third party data, please ensure that the statement adheres to our policy

All data generated or analysed during this study are included in the paper and its Supplementary Information. Source data are provided as a Source Data file.

Research involving human participants, their data, or biological material

Policy information about studies with human participants or human data. See also policy information about sex, gender (identity/presentation), and sexual orientation and race, ethnicity and racism.

Reporting on sex and gender	N/A
Reporting on race, ethnicity, or other socially relevant groupings	N/A
Population characteristics	N/A
Recruitment	N/A
Ethics oversight	N/A

Note that full information on the approval of the study protocol must also be provided in the manuscript.

Field-specific reporting

Please select the one below that is the best fit for your research. If you are not sure, read the appropriate sections before making your selection.

☒ Life sciences ☐ Behavioural & social sciences ☐ Ecological, evolutionary & environmental sciences

For a reference copy of the document with all sections, see nature.com/documents/nr-reporting-summary-flat.pdf

Life sciences study design

All studies must disclose on these points even when the disclosure is negative.

Sample size	Experiments were performed and repeated independently. Mean, standard error of the mean and standard deviation are calculated as described below in 'Replication'. We collected as much experimental data as possible. To ensure reproducibility, a minimum of three biological replicates were conducted. We observed low variability and performed sample size test, confirming our sample size to be sufficient.
Data exclusions	No data are excluded from the analysis.
Replication	Data in text are presented as mean ± standard error of the mean (S.E.M.). Data in figures are presented either as mean ± S.E.M. (Fig. 2d,e, Fig. 3g,i, Fig. 4 g,i, Fig. 5b and Extended Data Fig. 6d) or mean ± Standard Deviation (SD) range (Fig. 5d). For Fig. 2d,e and Fig. 3g,i, biological replicates n = 3. For Fig. 4k, biological replicates n ≥3. For Fig. 4g, biological replicates n ≥4. For Fig. 4i, biological replicates n ≥5. For Fig. 5b,d, biological replicates n ≥3. For Fig. 5o, biological replicates n = 3. Statistical analysis was performed using GraphPad Prism 9. A detailed statistical analysis is listed in Supplementary Table 4.
Randomization	Randomization methods did not apply to this study as there were no clinical populations or patients involved. All cells, printed tissues, and brain slices that successfully passed the quality filter were utilized for downstream applications and analysis. Regarding microscopic imaging, a minimum of three random areas were taken for subsequent analysis.
Blinding	The researcher were not blinded during experiments. As our measurement are based on objective data (e.g. process outgrowth and migration visualized by fluorescence labelled cells), blinding does not affect these data values.

Reporting for specific materials, systems and methods

We require information from authors about some types of materials, experimental systems and methods used in many studies. Here, indicate whether each material, system or method listed is relevant to your study. If you are not sure if a list item applies to your research, read the appropriate section before selecting a response.

Materials & experimental systems

n/a	Involved in the study
☐	☒ Antibodies
☐	☒ Eukaryotic cell lines
☒	☐ Palaeontology and archaeology
☐	☒ Animals and other organisms
☒	☐ Clinical data
☒	☐ Dual use research of concern
☒	☐ Plants

Methods

n/a	Involved in the study
☒	☐ ChIP-seq
☒	☐ Flow cytometry
☒	☐ MRI-based neuroimaging

Antibodies

Antibodies used

Primary Antibodies
Target; Original Species; Manufacturer; Dilution Factor; Cat. No

CUX1 Ms Santa Cruz Biotechnology 100 sc-13024
CUX2 Rb AbCam 200 ab216588
BRN2 Ms Santa Cruz Biotechnology 100 sc-393324
SATB2 Rb AbCam 200 ab92446
CTIP2 Rat AbCam 200 ab18465
TBR1 Rb Merck 500 AB10554
SOX2 Rb Millipore 100-200 ab5603
TUJ1 Ms AbCam 500-1000 ab78078
HNCAM Rb AbCam 200 ab75813

Secondary Antibodies
Target Species; Original Species; Fluorophore; Manufacturer; Dilution Factor; Cat. No
Rb Goat Alex488 Invitrogen 1000 a11006
Ms Goat Alex488 Invitrogen 1000 a32723
Ms Goat Alex633 Invitrogen 1000 a21052
Rat Goat Alex647 Invitrogen 1000 a21247
Rb Goat Alex647 Invitrogen 1000 a21245

Validation

sc-13024:https://www.scbt.com/p/cdp-antibody-m-222
ab216588, species reactivity: Mouse, Rat and Human, https://www.abcam.com/cux2-antibody-ab216588.html
sc-393324, species reactivity: Mouse, Rat and Human, https://www.scbt.com/p/brn-2-antibody-b-2
ab92446, species reactivity: Mouse, Rat and Human, https://www.abcam.com/satb2-antibody-epncir130a-ab92446.html
ab18465, species reactivity: Mouse, Human, https://www.abcam.com/ctip2-antibody-25b6-ab18465.html
AB10554, species reactivity: Mouse, Rat, Horse etc, https://www.merckmillipore.com/GB/en/product/Anti-Tbr1-Antibody,MM_NF-AB10554?ReferrerURL=https%3A%2F%2Fwww.google.com%2F
ab5603, species reactivity: Human, Mouse, https://www.merckmillipore.com/GB/en/product/Anti-Sox2-Antibody,MM_NF-AB5603?ReferrerURL=https%3A%2F%2Fwww.google.com%2F
ab78078, species reactivity: Mouse, Rat, Human etc, https://www.abcam.com/beta-iii-tubulin-antibody-2g10-neuronal-marker-ab78078.html
ab75813, species reactivity: Human, https://www.abcam.com/ncam1-antibody-ep2567y-ab75813.html

Validation information of secondary antibodies can be found at ThermoFisher's Websites.

Eukaryotic cell lines

Policy information about cell lines and Sex and Gender in Research

Cell line source(s)

The iPSC line (AH016-3) used in this study was provided by Dr Sally Cowley (James Martin Stem Cell Facility, Oxford). A detail description can be found at Dr Sally Cowley's publication (doi: 10.1016/j.stemcr.2017.05.017).

Authentication

The iPSC lines were authenticated in James Martin Stem Cell Facility, Oxford. The AH016-3 cell line was published in PMID:28096185 and the characterisation of the line is given in the supplementary figures of that paper, which comprises:

- Transgene silencing in iPSC lines assessed by qPCR.
- Genome integrity was assessed by illumina Human CytoSNP-12v2.1 SNP array.
- Tra-1-60 Pluripotency protein expression, as shown by FACs.
- iPSC colony morphology, with high nucleus to cytoplasm ratio by phase-contrast microscopy.
- PluriTest analysis of illumina HT12v4 transcriptome array data.

The introduction of RFP into the above line (AH016-3 Lenti_RFP_IP (11 copy)) was published in PMID: 28591653. The

	methodology for introduction of the RFP transgene is given in the supplementary information of that paper.
Mycoplasma contamination	The hiPSC lines were mycoplasma tested negative in Martin Stem Cell Facility.
Commonly misidentified lines (See ICLAC register)	No Commonly misidentified lines are used.

Animals and other research organisms

Policy information about studies involving animals; ARRIVE guidelines recommended for reporting animal research, and Sex and Gender in Research

Laboratory animals	P8-10 C57BL/6 mice. All mice used in this study were maintained in pathogen-free facilities at the University of Oxford. Mice were given ad libitumaccess to food and water.
Wild animals	No wild animals were used.
Reporting on sex	Sex was not considered in this study as only animal tissues were used.
Field-collected samples	No field-collected samples were used in this study.
Ethics oversight	Animals Scientific Procedures Act (1986) in United kingdom with licence no. PP8557407

Note that full information on the approval of the study protocol must also be provided in the manuscript.
